# Systemic and Cardiac Microvascular Dysfunction in Hypertension

**DOI:** 10.3390/ijms252413294

**Published:** 2024-12-11

**Authors:** Alessandro Durante, Alessandro Mazzapicchi, Martina Baiardo Redaelli

**Affiliations:** 1 Interventional and Clinical Cardiology Unit, Policlinico San Marco, 24040 Zingonia, Italy; 2 Azienda Ospedaliero-Universitaria Policlinico “Sant’Orsola”, University of Bologna, 40125 Bologna, Italy; alessand.mazzapicchi@studio.unibo.it; 3 Dipartimento di Biotecnologie e Scienze della Vita, ASST Sette Laghi, Università degli Studi dell’Insubria, 21100 Varese, Italy; m.baiardoredaelli@uninsubria.it

**Keywords:** microvascular, hypertension, heart, brain

## Abstract

Hypertension exerts a profound impact on the microcirculation, causing both structural and functional alterations that contribute to systemic and organ-specific vascular damage. The microcirculation, comprising arterioles, capillaries, and venules with diameters smaller than 20 μm, plays a fundamental role in oxygen delivery, nutrient exchange, and maintaining tissue homeostasis. In the context of hypertension, microvascular remodeling and rarefaction result in reduced vessel density and elasticity, increasing vascular resistance and driving end-organ damage. The pathophysiological mechanisms underlying hypertensive microvascular dysfunction include endothelial dysfunction, oxidative stress, and excessive collagen deposition. These changes impair nitric oxide (NO) bioavailability, increase reactive oxygen species (ROS) production, and promote inflammation and fibrosis. These processes lead to progressive vascular stiffening and dysfunction, with significant implications for multiple organs, including the heart, kidneys, brain, and retina. This review underscores the pivotal role of microvascular dysfunction in hypertension-related complications and highlights the importance of early detection and therapeutic interventions. Strategies aimed at optimizing blood pressure control, improving endothelial function, and targeting oxidative stress and vascular remodeling are critical to mitigating the systemic consequences of hypertensive microvascular damage and reducing the burden of related cardiovascular and renal diseases.

## 1. Introduction

The microcirculation represents the distal vascular bed of the systemic circulation, comprising vessels with diameters < 20 μm, including arterioles, capillaries, and venules. Its primary function is to ensure oxygen delivery to parenchymal cells to support their activity. Additionally, microcirculation regulates solute exchange between the intravascular and tissue spaces and facilitates the delivery of hormones and nutrients to cells. The microvasculature is predominantly lined by endothelial cells (ECs), which, despite varying across organs and tissues, work in concert with smooth muscle cells to regulate blood flow. The regulation of microvascular blood flow is mediated by myogenic, metabolic, and neurohumoral mechanisms [1].

Microcirculation has been shown to be affected by several acute and chronic diseases, among which hypertension is particularly significant due to its prevalence and its widespread impact on multiple organs. Hypertension affects 30–50% of the population in Western countries, making it one of the most common diseases. The first description of microcirculatory abnormalities in hypertensive patients was provided by Richard Bright, a nephrologist, in the early 19th century [2]. In 1869, George Johnson offered histological evidence of wall thickening in small arteries [3], and, shortly after, Ewald suggested that high blood pressure (BP) might be a cause, rather than a consequence, of microvascular alterations [4]. Essential hypertension was later recognized as an independent disease during the early 20th century [5].

Hypertension induces two primary structural changes in the systemic microcirculation: rarefaction, a reduction in vessel density, and remodeling, structural modifications of resistance small arteries and arterioles. The concept of “vascular remodeling” was introduced in 1959, though the relationship between vascular changes and specific conditions had been acknowledged since the mid-20th century. While remodeling may also occur in large arteries, arterial hypertension is predominantly associated with microcirculatory alterations [6].

In essential hypertension, remodeling often involves a narrowing of the internal lumen and an increase in the thickness of the tunica media or total vessel wall. This results in an elevated media-to-lumen ratio (MLR) or wall-to-lumen ratio (WLR) [5,6]. Typically, the increased MLR in hypertension is attributed to inward eutrophic remodeling, where normal material rearranges around a narrowed lumen. Conversely, inward hypertrophic remodeling, characterized by vascular smooth muscle cell hypertrophy or hyperplasia, has been observed in patients with type II diabetes mellitus, obesity, and metabolic syndrome, irrespective of hypertension [7,8].

## 2. Vascular Changes

**Figure 1:**
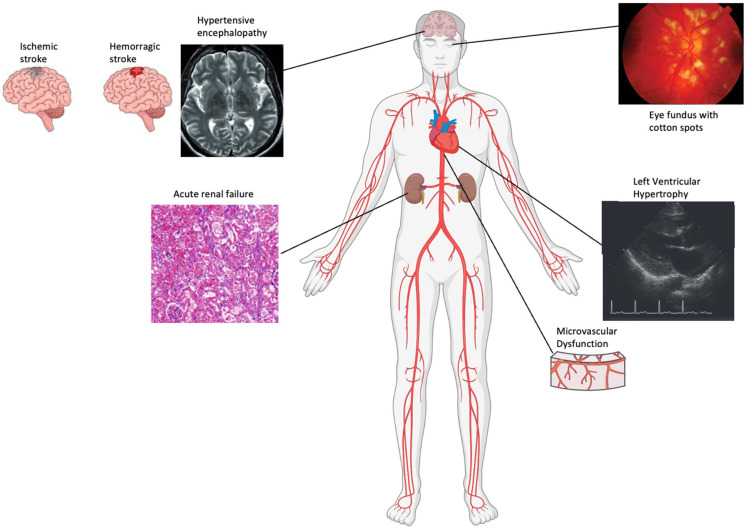
Complications specific to uncontrolled hypertension. (**top left**) Hypertensive encephalopathy with PRES on MRI, ischemic and hemorrhagic stroke; (**top right**) retinal photograph showing cotton wool spots; (**left down**) a kidney biopsy in a patient with acute kidney injury with hematuria and proteinuria; (**right down**) left ventricular hypertrophy in patients with hypertensive cardiopathy with associated microvascular disfunction [9,10,11,12].

Under both normal and pathological conditions, a pressure drop occurs between large arteries and capillaries, with most of it taking place in resistance vessels. Although their exact location lacks a precise definition (Figure 1) [9,10,11,12], resistance vessels are generally identified as small arteries and arterioles with diameters ranging from 300 to 15 μm [13]. These vessels are characterized by their ability to exhibit myogenic tone, which refers to their contraction in response to increases in transmural pressure [13]. Notably, the myogenic tone becomes more pronounced as vessel diameter decreases [14].

Two mechanisms of remodeling—eutrophic and hypertrophic—have been discussed, but their distinct pathways merit clarification. Eutrophic remodeling, primarily observed in small resistance arteries, involves a rearrangement of vascular smooth muscle cells (VSMCs) around a narrowed lumen without any net increase in wall mass. In contrast, hypertrophic remodeling, which is more prominent in metabolic disorders like diabetes mellitus, leads to an increase in wall mass due to VSMC hyperplasia. Both types of remodeling affect vascular tone and result in increased wall-to-lumen ratios, playing a significant role in the pathophysiology of hypertension [15].

The structural properties of blood vessels under physiological and pathological conditions can be analyzed using Laplace’s Law, which relates intramural stress (s), wall thickness (W), lumen radius (r), and transmural pressure (P, the difference between luminal and extraluminal pressures). The law is expressed as

s = Pr/W(1)

which, in terms of the W-to-lumen diameter (D) ratio, can be presented as

s = 0.5(P/(W/D))(2)

Folkow was the first to propose that structural alterations could lead to increased vascular resistance, even in the absence of elevated vascular tone [5]. According to Poiseuille’s Law, vascular resistance is inversely proportional to the fourth power of the radius (r^4^). Consequently, even small reductions in the lumen diameter (D) can significantly increase resistance. In hypertension, microvascular remodeling with wall thickening leads to an increased wall-to-lumen diameter ratio (W/D) by structurally reducing D. Under such conditions, the same level of vascular tone and smooth muscle cell (SMC) contraction causes a greater reduction in D, simply due to geometrical constraints. Folkow’s concepts have formed the foundation for modern theories on the pathogenesis of hypertension. It is now widely accepted that while vascular tone acts as a short-term regulator, remodeling is the key determinant of long-term hypertension [16,17,18].

## 3. Molecular Mechanism

The production of reactive oxygen species (ROS) plays a pivotal role in the development of vascular dysfunction, particularly in hypertension. ROS are generated through several enzymatic pathways, including the NADPH oxidase complex, mitochondrial dysfunction, and the uncoupling of endothelial nitric oxide synthase (eNOS) [19]. When ROS production surpasses the body’s antioxidant capacity, oxidative stress occurs, leading to cellular and vascular damage [19]. A key consequence of oxidative stress is the reduction in the availability of nitric oxide (NO), a critical molecule for maintaining vascular homeostasis through vasodilation. Under normal conditions, endothelial cells synthesize NO via eNOS, enabling the relaxation of blood vessels. However, excessive ROS, particularly superoxide anions, react with NO to form peroxynitrite (ONOO^−^), a highly reactive and damaging compound. This interaction reduces NO bioavailability, impairing endothelial-dependent vasodilation and increasing vascular resistance and blood pressure [19]. This diminished vasodilatory response is a hallmark of endothelial dysfunction, a precursor to atherosclerosis and other cardiovascular diseases [19].

**Figure 2:**
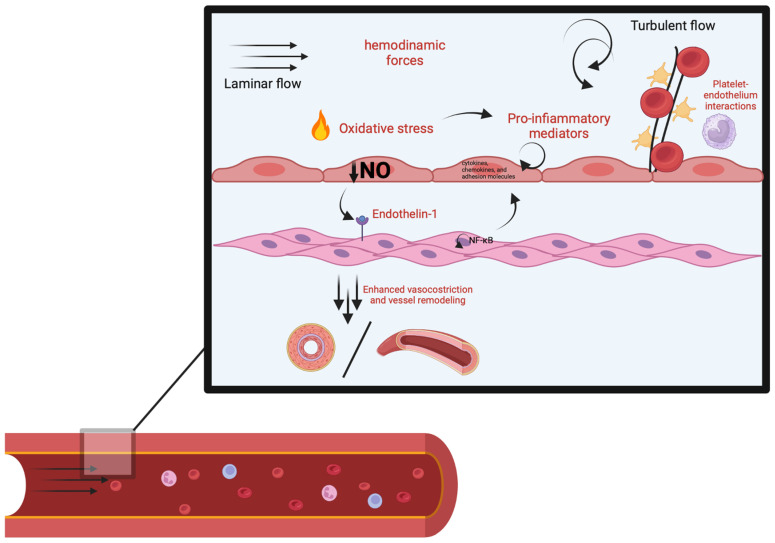
Molecular mechanism of microvascular dysfunction: turbulent flow and other pro-inflammatory factors led to a reduction in intracellular NO, causing different self-perpetuating cycles that increase vasoconstriction and vessel remodeling.

In addition to reducing NO levels, oxidative stress promotes vascular inflammation by activating transcription factors such as nuclear factor kappa B (NF-κB). This activation leads to the upregulation of pro-inflammatory cytokines, chemokines, and adhesion molecules, further damaging the endothelium and exacerbating vascular dysfunction (Figure 2) [19].

Another key feature of hypertensive vascular pathology is the dysregulation of collagen, particularly type I collagen, which provides structural support to blood vessels. In hypertensive patients, there is an overproduction of type I collagen, resulting in increased vessel stiffness and fibrosis [20]. This excessive collagen deposition occurs mainly in the extracellular matrix (ECM) and is driven by fibrogenic pathways, such as the activation of transforming growth factor-beta (TGF-β) [21].

Furthermore, mechanosensing molecular mechanisms in endothelial and vascular smooth muscle cells (VSMCs) contribute significantly to vascular remodeling. Phase transitions of stressed endothelial cells lead to the expression of contact proteins, facilitating the rolling, marginalization, and migration of white blood cells, which amplify inflammatory responses [22]. In VSMCs, phase transitions are associated with the release of pro-inflammatory cytokines and the expression of matrix metalloproteinases (MMPs). MMPs degrade elastic fibers and promote fibrosis, which contributes to vascular stiffening and remodeling [22].

The apoptosis of VSMCs, supported by well-known intracellular signaling pathways, further exacerbates vascular damage. Among these, the TGF-β pathway plays a central role by promoting fibrotic changes and stimulating the overproduction of type I collagen [22]. Additionally, the intracellular signaling processes linked to ROS and the activation of angiotensin II receptors intensify these fibrotic pathways. Angiotensin II, a key mediator in hypertension, triggers both oxidative stress and inflammation, reinforcing the vicious cycle of vascular injury [19,20,21,22].

The fibrotic remodeling of the vascular wall caused by collagen overproduction results in a loss of vessel elasticity, contributing to elevated systemic vascular resistance and impaired hemodynamic adaptability [20]. This stiffening process not only affects large vessels but also disrupts the function of small arteries and arterioles, which are critical for regulating blood flow and pressure [20]. The progressive fibrosis of the microvasculature reduces its capacity to respond to physiological changes, thereby exacerbating hypertension and contributing to end-organ damage in systems such as the heart, kidneys, and brain [20].

The combination of oxidative stress and collagen dysregulation creates a vicious cycle involving endothelial dysfunction, inflammation, and fibrosis. This cycle leads to progressive vascular stiffening and dysfunction, hallmark features of hypertensive vascular disease. As oxidative stress impairs NO bioavailability and promotes inflammation, the resulting endothelial injury accelerates fibrotic changes in the vascular wall. This, in turn, perpetuates vascular stiffness, further increasing blood pressure and contributing to the pathogenesis of hypertension [20,21,22].

## 4. Methods to Evaluate Vascular Structural Alterations

Assessing microvascular alterations is inherently challenging, with relatively few methods available. Traditional histological approaches are widely used but have significant limitations. Artifacts introduced during tissue fixation, staining, and dehydration can lead to distortions such as coarctation, reducing the reliability of results [6]. Consequently, more advanced and precise techniques have been developed to evaluate microcirculation both structurally and functionally.

One such method is plethysmography, which measures minimum vascular resistance in the forearm. This is achieved by calculating the maximum post-ischemic blood flow under conditions of maximum vasodilation, providing an indirect index of microvascular structural alterations. The approach offers the advantage of an in vivo assessment, albeit indirectly, linking resistance changes to the wall-to-lumen ratio (WLR), a critical parameter in understanding microvascular remodeling [23,24].

A more direct and widely applied method for evaluating peripheral microcirculation is wire or pressure micromyography, which allows reliable analysis of both structural and functional aspects of small resistance arteries. A particularly valuable parameter derived from this technique is the media-to-lumen ratio (MLR), which is independent of vessel dimensions and provides robust insights into microvascular alterations [25,26]. Comparative studies between plethysmography and micromyography in both normotensive and hypertensive populations have demonstrated a strong correlation between the MLR of subcutaneous small arteries and forearm minimum vascular resistance. These findings underscore the complementarity of the two methods, as micromyography excels in assessing precise morphological changes but requires tissue samples obtained during surgery or through subcutaneous fat biopsy, while plethysmography offers a non-invasive, functional evaluation [27].

Another approach for studying peripheral microvasculature is capillary microscopy or capillaroscopy, which has shown significant reductions in capillary density, such as the 20% decline observed in the nailfold capillaries of hypertensive patients [28]. Similar patterns of reduced arteriolar and capillary density have been detected in the conjunctival microcirculation using intravital videomicroscopy, an in vivo visualization technique [29]. Capillaroscopy provides a two-dimensional view of the capillary network and enables the assessment of capillary morphology, density, flow velocity, and red blood cell column width. The use of fluorescent dyes enhances its capabilities by allowing the evaluation of capillary flow heterogeneity and permeability, and identifying pathological structures like aneurysms. Moreover, this technique has demonstrated the presence of microvascular rarefaction in obese individuals independently of their blood pressure levels, highlighting its utility in broader clinical contexts [30,31].

A novel development in this field is the use of handheld vital microscopes (HVMs), primarily for studying sublingual microcirculation. Alterations in sublingual microvascular parameters have shown high sensitivity and specificity in predicting morbidity and mortality across various clinical conditions, further solidifying its role in microvascular research [32,33].

The assessment of coronary microcirculation presents additional challenges due to the lack of direct in vivo visualization techniques for humans. Indirect methods for evaluating coronary microvascular function rely on measuring blood flow. Techniques such as intracoronary thermodilution and the use of intracoronary Doppler wires have proven effective for quantifying flow rates. More recently, Doppler echocardiography has emerged as a non-invasive alternative for assessing coronary blood flow [34,35]. These methods, however, primarily measure total coronary blood flow without isolating the contribution of the microvascular network.

The TIMI myocardial perfusion grade offers another approach, relying on the relative intensity and clearance speed of myocardial “blush” observed during coronary angiography following contrast injection. This visual estimation correlates the functionality of the coronary microvasculature with perfusion scores ranging from 0 to 3, with higher scores indicating better perfusion. However, while TIMI grading is a valuable tool, it is inherently subjective and less precise compared to advanced imaging modalities [36,37].

Positron emission tomography (PET) offers a more quantitative approach by calculating blood flow per unit of tissue mass, thereby providing detailed insights into microvascular function [34]. Similarly, cardiac magnetic resonance (CMR), particularly with contrast enhancement, demonstrates superior accuracy in detecting microvascular obstruction post-myocardial infarction and correlates strongly with adverse cardiac events. These imaging techniques provide a significant advantage over traditional methods, offering more precise assessments of coronary microvascular function [38,39,40].

Finally, the concept of coronary flow reserve (CFR) provides a critical parameter for evaluating coronary microcirculation. CFR measures the ability of the microvascular bed to increase blood flow in response to maximal vasodilation, reflecting its functional reserve. This parameter is particularly valuable in patients without epicardial coronary artery stenoses, as it isolates the contribution of the microvasculature to coronary flow dynamics. In the presence of epicardial coronary artery disease (CAD), however, CFR is influenced by the severity of the stenosis, making the assessment of microvascular dysfunction more complex due to the interplay of various clinical and hemodynamic factors [34].

In summary, the evaluation of microvascular alterations requires a combination of structural and functional techniques tailored to the specific vascular bed of interest. While traditional methods like plethysmography and micromyography remain foundational, advances in imaging and visualization, such as PET, CMR, and capillaroscopy, continue to expand our understanding of microvascular health and dysfunction in both peripheral and coronary circulation. These approaches provide critical insights into the pathophysiology of conditions such as hypertension, obesity, and cardiovascular disease, enabling more targeted diagnostic and therapeutic strategies.

### 4.1. Kidney

The renal microcirculation consists of interlobar, arcuate, and interlobular arteries. Beyond this point, it differs significantly from the microcirculation in other organs. The afferent arterioles give rise to the glomerular capillaries, where plasma filtration occurs. Following the glomerulus, blood exits through the efferent arterioles, which then branch into two distinct pathways. The descending vasa recta, originating from the efferent arterioles, carry arterial blood into the renal medulla and transition into the ascending vasa recta, which carry venous blood. In addition, the efferent arterioles form peritubular capillary networks, ensuring the reabsorption of essential materials previously filtered and returning them to the circulation. This capillary network ultimately drains into the venules.

Renal microcirculation is tightly regulated by tubuloglomerular feedback, which adjusts afferent arteriole tone in response to changes in renal perfusion pressure and sodium levels; meanwhile, the constriction or dilation of efferent arterioles is primarily influenced by angiotensin II. This intricate autoregulatory mechanism maintains glomerular filtration and protects the kidney from fluctuations in systemic blood pressure (BP).

Arterial hypertension is a leading cause of chronic kidney disease (CKD) [41]. Damage to preglomerular arteries and arterioles results in ischemia and the progressive narrowing of the preglomerular microcirculation. Simultaneously, the inability of the kidney to properly regulate blood flow allows elevated systemic BP to be transmitted to the glomeruli, causing hyperperfusion and glomerular hypertension. This leads to structural injury, including glomerulosclerosis, and the progressive decline of renal function [42]. Hypertension not only contributes to CKD development but also makes the kidney a primary target of hypertensive damage.

Hypertensive damage to the renal microcirculation is central to CKD progression [43]. Microvascular remodeling and rarefaction reduce perfusion, leading to ischemia and tubular injury. The impaired autoregulation in hypertensive kidneys exacerbates glomerular hyperfiltration and fosters further glomerular damage. These changes culminate in progressive renal impairment, a frequent outcome in individuals with uncontrolled hypertension. Notably, untreated hypertension results in proteinuria in up to 42% of patients and advanced renal failure in 18% [39]. With an aging population and the increasing prevalence of hypertension and diabetes, CKD affects a substantial portion of the general population, with 6–7% exhibiting high albuminuria and a preserved glomerular filtration rate (GFR), and 3–5% presenting with a GFR below 60 mL/min/1.73 m² [44,45].

Even treated hypertension remains a significant cause of CKD, second only to diabetic nephropathy as a cause of end-stage renal disease [42]. Hypertension promotes CKD via a combination of functional and structural changes in the renal microcirculation. Vascular rarefaction, the reduction in the number of arterioles and capillaries, is the final consequence of prolonged exposure to elevated renal perfusion pressure. This process is preceded by vascular remodeling, which is driven by endothelial dysfunction—a key factor in CKD progression in hypertensive patients [46].

The concept of microvascular rarefaction was first described in the 1970s in animal models of hypertension [47]. Studies in spontaneously hypertensive rats demonstrated increased vascular resistance and functional rarefaction, which was later followed by structural rarefaction, characterized by the loss of terminal arterioles and capillaries [48,49]. These findings have since been replicated in human hypertension [50]. Rarefaction contributes to reduced renal tissue perfusion and oxygenation, exacerbating target organ damage [51].

The high systemic BP transmitted to the renal circulation is not the sole factor driving kidney disease. In response to hypertension, the kidney releases angiotensin II and endothelin-1, both of which exacerbate microvascular damage. Studies have shown that blocking these pathways in hypertensive models significantly reduces microvascular rarefaction [52,53]. Both angiotensin II and endothelin-1 suppress nitric oxide (NO) release and activity, increasing inflammation, fibrosis, and vascular remodeling. These effects are mediated directly and indirectly through the generation of reactive oxygen species [54].

The blockade of the renin–angiotensin system (RAS) using angiotensin-converting enzyme inhibitors (ACEIs) or angiotensin receptor blockers (ARBs) is a cornerstone of hypertension treatment, protecting both the macrocirculation and microcirculation [55]. Early-stage hypertension studies in humans have shown that RAS blockade can improve alterations in the WLR of resistance arterioles, which contribute to the increased peripheral resistance characteristic of hypertension [56].

To summarize, the renal microcirculation plays a critical role in the development and progression of CKD in the context of hypertension. The combination of microvascular remodeling, rarefaction, and endothelial dysfunction drives ischemia and progressive renal impairment. Understanding these mechanisms underscores the importance of effective BP control and early intervention with therapies targeting the RAS to mitigate CKD progression and reduce the burden of hypertensive kidney damage.

### 4.2. Brain

The cerebral vasculature is a major target of hypertensive damage, with hypertension inducing significant structural changes in the vascular wall [57,58]. Within the cerebrovascular tree, hypertension is strongly associated with arterial stiffening [59,60], which amplifies pulse pressure and mechanical stress. These changes prompt adaptive responses designed to protect the downstream microcirculation, leading to vascular remodeling of vascular smooth muscle cells (VSMCs) driven by mechanical, cellular, and molecular factors [61]. This remodeling often manifests as an increase in the number and volume of VSMCs without changes in cross-sectional area, resulting in reduced luminal diameter and an elevated wall-to-lumen ratio (WLR).

Hypertension-induced vascular remodeling has been demonstrated in cerebral small vessels [58]. Moreover, arterial stiffness has been linked to cerebral small vessel disease (SVD), cognitive decline, and dementia. Remodeling in the cerebral microcirculation arises from the interplay of mechanical, cellular, and molecular mechanisms, with angiotensin II playing a prominent role through its growth-promoting and pro-inflammatory effects. Angiotensin II, a key peptide in hypertension pathophysiology, also increases the production of reactive oxygen species (ROS), exacerbating vascular injury [61,62,63].

Hypertension is a major risk factor for macrovascular damage in the brain, including atherosclerosis and stroke, but also significantly impacts the microvasculature. Evidence of microvascular rarefaction has been observed in both human and animal models of hypertension [60,61]. In white matter (WM), where vessel density is relatively low, hypertensive rarefaction may contribute to WM lesions. This process is believed to result from elevated pressure transmitted to the microvascular bed, although the precise mechanisms remain unclear [64]. Typical hypertensive microvascular lesions include lipohyalinosis (the deposition of a glass-like material in vessel walls) and fibrinoid necrosis, particularly in the WM arterioles [63].

Hypertension is a recognized cause of SVD, particularly in subcortical and periventricular WM arterioles, capillaries, and venules [65]. SVD is a major contributor to cognitive impairment and is characterized on MRI by WM hyperintensities (WMHs), lacunes (WM lesions < 15 mm), microhemorrhages, microinfarcts, and venous collagenosis [66,67]. Cerebral microhemorrhages, closely linked to hypertension, have been associated with worse cognitive outcomes [65,66,67,68,69,70]. The progression of WMHs is strongly related to the duration of hypertension and can be mitigated by effective BP control [66].

SVD is also associated with enlarged perivascular spaces (PVSs), suggesting the impaired clearance of potentially toxic proteins and metabolites from the brain [71]. PVSs’ involvement in clearance mechanisms remains a topic of debate, but studies in hypertensive rats and small patient cohorts have demonstrated an impaired cerebrospinal and interstitial fluid flow, contributing to metabolic waste accumulation [72,73,74,75]. The extent to which these clearance dysfunctions influence SVD pathobiology and cognitive deficits in hypertension is still under investigation.

The brain relies on stable and continuous blood delivery, given its limited energy reserves. Cerebrovascular autoregulation is a protective mechanism that maintains constant cerebral blood flow (CBF) despite fluctuations in BP, typically within ±20 mmHg of baseline [76]. Hypertension alters autoregulation, as demonstrated in animal studies, where the static pressure–flow curve shifts to the right [77]. This increases the risk of ischemia during BP drops. Structural changes such as arterial stiffening and VSMC remodeling, combined with heightened myogenic tone, contribute to these shifts. Importantly, studies have shown that antihypertensive therapy can reverse this rightward shift, suggesting that reducing vascular mechanical stress can restore autoregulatory function [78].

In humans, the precise impact of hypertension on the limits of autoregulation is less clear. While most studies report no significant rightward shift in the static autoregulatory curve, one small study demonstrated a shift in the lower limit of cerebral autoregulation [79]. Notably, antihypertensive treatment has been shown to maintain or even increase CBF, challenging earlier concerns that lowering BP could lead to cerebral hypoperfusion, particularly in older adults [80,81].

The endothelium plays a critical role in regulating vasomotor tone in the brain, as in other organs [80]. Hypertension impairs endothelial function, reducing nitric oxide (NO) availability and NO-mediated vasodilation. These effects are attributed to impaired endothelial nitric oxide synthase (eNOS) function, reduced NO production, and heightened vascular oxidative stress. Although a direct evaluation of cerebral endothelial function in humans is difficult, studies suggest impaired NO signaling. For instance, the use of NO synthesis inhibitors in hypertensive patients failed to reduce retinal arteriolar blood flow, indicating alterations in NO pathways and endothelial dysfunction [82]. Endothelial dysfunction in systemic arteries has been associated with WMHs and microhemorrhages, further supporting its role in cerebral microvascular pathology [83,84]. Postmortem studies of arterioles from SVD patients have shown impaired endothelium-dependent vasodilator responses to acetylcholine, reinforcing this hypothesis [85].

In severe cases of uncontrolled hypertension, brain involvement can manifest as hypertensive encephalopathy, observed in approximately 10% of patients with associated retinal abnormalities. Even without overt neurological symptoms, MRI can reveal findings such as posterior reversible encephalopathy syndrome (PRES). This condition often presents with altered consciousness, including delirium or slowed mental activity (bradyphrenia), and may progress to seizures or loss of consciousness. The most serious complications are cerebral hemorrhage and intracranial herniation. Early recognition and treatment of PRES generally lead to radiological and clinical reversibility, with a favorable prognosis.

### 4.3. Retina

One of the most clinically significant complications of microcirculatory damage in hypertension is hypertensive retinopathy, a condition marked by endothelial dysfunction and smooth muscle cell remodeling within the retinal arterioles. This pathology is initiated by prolonged elevated blood pressure, which imposes sustained mechanical stress on vascular walls, leading to damage of vascular endothelial cells. This disruption compromises the delicate balance between vasodilation and vasoconstriction, resulting in the impaired regulation of retinal blood flow [86].

A defining feature of hypertensive retinopathy is increased vascular permeability, stemming from the breakdown of the blood–retinal barrier. This loss of barrier integrity permits proteins and fluids to leak into the retinal tissues, causing retinal edema and hemorrhages [87]. These changes further undermine the retinal microvasculature, promoting ischemia and heightened oxidative stress. The remodeling of smooth muscle cells within retinal arterioles, coupled with endothelial dysfunction, exacerbates the condition by increasing vascular stiffness and resistance.

Although hypertensive retinopathy is often asymptomatic in its early stages, the progressive nature of retinal damage can eventually lead to significant visual impairment. In advanced stages, complications such as retinal ischemia, hard exudates, and, in severe cases, optic nerve damage can occur, potentially resulting in irreversible vision loss [88,89]. These pathological changes highlight the critical need for early detection and intervention.

Given the systemic impact of hypertension, which affects multiple organ systems, the retina often serves as a valuable indicator of broader microvascular damage throughout the body. As such, early identification of retinal changes in hypertensive patients can provide an important marker for potential cardiovascular and renal complications, emphasizing the role of hypertensive retinopathy as both a localized and systemic manifestation of microvascular dysfunction.

### 4.4. Heart

Coronary microvascular dysfunction (CMD) is highly prevalent in hypertensive patients, affecting up to 50% of this population. Hypertension contributes to CMD by inducing endothelial dysfunction and increasing oxidative stress, which impair endothelial cell function [90]. These changes disrupt nitric oxide (NO) production and release while increasing the secretion of endothelin-1 (ET-1). In addition to these functional impairments, hypertension causes structural alterations in the coronary microvasculature, including vessel wall thickening, fibrosis, and smooth muscle cell hypertrophy.

Patients with CMD and hypertension are at an elevated risk of adverse cardiovascular events such as myocardial infarction, heart failure, and sudden cardiac death. A significant number of patients undergoing coronary angiography for anginal symptoms or a positive stress test are found to have no flow-limiting stenosis in the epicardial arteries [88]. These cases are classified as angina with no obstructive coronary artery disease (ANOCA) or ischemia with no obstructive coronary artery disease (INOCA) [91]. INOCA is associated with increased morbidity and mortality, including conditions like myocardial infarction with no obstructive coronary artery disease (MINOCA) and heart failure with preserved ejection fraction (HFpEF) [92].

The pathophysiology of CMD differs from that of epicardial flow-limiting stenoses, leading to distinct clinical presentations. In epicardial stenosis, myocardial perfusion impairment is localized to the vascular territory supplied by the affected artery, resulting in segmental wall motion abnormalities. In contrast, CMD causes patchy ischemia due to the heterogeneous involvement of microvessels, often without associated myocardial wall contraction abnormalities. This diffuse and irregular ischemia can manifest as INOCA or MINOCA, with symptoms and elevated myocardial damage markers despite the absence of significant epicardial stenosis [93].

Several studies have demonstrated that coronary flow reserve (CFR) reduction in hypertensive patients occurs independently of the presence or degree of left ventricular hypertrophy (LVH), suggesting that CMD arises primarily from functional and structural microvascular alterations [94]. Nevertheless, hypertensive heart disease is largely driven by the myocardial response to elevated blood pressure [95,96].

In patients with uncontrolled hypertension, transthoracic echocardiography often reveals an increase in left ventricular (LV) mass, progressive hypertrophy, and an enlargement of the LV, left atrium, and aortic root. Additionally, a reduction in LV ejection fraction may occur. These structural changes are more pronounced compared to those observed in patients with well-managed essential hypertension. Advanced imaging techniques, such as speckle tracking echocardiography and cardiac magnetic resonance (CMR), have provided insights into the severity and progression of cardiac damage in LVH. For instance, global longitudinal strain analysis has shown reduced LV longitudinal fiber function in patients with uncontrolled hypertension [97,98,99]. CMR is particularly valuable for identifying myocardial fibrosis, edema, and symmetrical LV hypertrophy. Following antihypertensive treatment, a regression of LV hypertrophy and improvements in systolic function have been observed, although residual hypertrophy and functional impairments often persist even with long-term BP control [98,99,100,101,102].

LVH in hypertension not only causes microvascular rarefaction but also leads to interstitial fibrosis and myocyte hypertrophy, increasing the intercapillary distance and reducing oxygen delivery to myocytes. The progression from hypertension to heart failure is not solely determined by the type of LV remodeling (adaptive or maladaptive) and may occur even in patients with normal LV geometry and function [103]. Interestingly, most patients with LVH progress directly to heart failure rather than following myocardial infarction, suggesting that CMD plays a pivotal role in the transition from hypertensive heart disease to left ventricular dysfunction or heart failure in the absence of epicardial coronary artery disease [104,105].

CMD may also be driven by a low-grade inflammatory response associated with hypertension, similar to mechanisms observed in obstructive coronary artery disease [106]. This inflammation reduces NO bioavailability, impairing both endothelium-dependent and endothelium-independent vasodilation. The resulting impaired vasomotor function increases the risk of myocardial ischemia. Importantly, this endothelial dysfunction may affect not only the coronary microcirculation but also the epicardial arteries and peripheral vasculature, reflecting a systemic disorder [107].

It has been proposed that inflammation-induced endothelial dysfunction may be mediated by elevated C-reactive protein (CRP) levels [108]. However, there remains debate over whether CRP is a causal factor in atherosclerosis, a consequence of the disease, or simply a confounding marker [109,110]. Further research is needed to clarify the precise role of CRP in the pathogenesis of CMD and its broader implications in hypertensive cardiovascular disease.

In summary, CMD in hypertension represents a multifaceted condition characterized by functional and structural microvascular alterations, systemic endothelial dysfunction, and chronic inflammation. These processes collectively contribute to increased cardiovascular risk and underscore the need for early detection and targeted therapeutic strategies to mitigate the progression to heart failure and other adverse outcomes.

## 5. Conclusions

The complex interplay between hypertension and microvascular dysfunction underscores the pivotal role of the microcirculation in systemic and organ-specific pathologies. Despite considerable advancements, significant knowledge gaps remain, offering opportunities for further exploration. One important avenue for future research is to clarify the molecular pathways linking microvascular rarefaction to systemic inflammation. The interplay between endothelial dysfunction, oxidative stress, and inflammatory mediators, including C-reactive protein and cytokines, suggests a systemic process that extends beyond localized vascular damage. A deeper understanding of these interactions could lead to innovative therapies aimed at reducing inflammation and preserving vascular integrity. The development and refinement of advanced imaging techniques present another promising direction. High-resolution imaging of the microcirculation, combined with non-invasive methods to assess functional changes, could enable earlier detection of vascular alterations. Standardizing these approaches and integrating them into clinical practice remains a priority. Moreover, research should focus on tools that monitor microvascular responses to antihypertensive therapies, paving the way for more personalized treatment strategies. The role of emerging biomarkers linked to endothelial health, oxidative stress, and vascular remodeling holds significant potential for identifying individuals at a greater risk of hypertensive complications. Future studies could explore how combining biomarker analysis with advanced imaging might improve risk stratification and inform targeted interventions. A particularly exciting frontier lies in the exploration of vascular regenerative therapies. Strategies such as the use of endothelial progenitor cells, gene therapies targeting nitric oxide pathways, and interventions to reverse vascular remodeling offer promising solutions for repairing damaged microcirculation. In summary, while much progress has been made in understanding how hypertension affects the microvasculature, there remain significant opportunities to refine diagnostic methods, identify novel therapeutic targets, and explore regenerative strategies. Advancing these research priorities is essential to reduce the burden of hypertension-related complications and improve outcomes for patients.

Table 1 summarizes the key features of microvascular dysfunction in hypertension. The table highlights the main processes involved, including oxidative stress, endothelial dysfunction, inflammation, ECM remodeling, VSMC dysfunction, the activation of the RAS, and rarefaction. ROS, generated through pathways such as NADPH oxidase, mitochondrial dysfunction, and the uncoupling of eNOS, contribute to oxidative stress and NO bioavailability, impairing vasodilation and promoting endothelial dysfunction. Inflammatory responses, driven by NF-κB, lead to endothelial activation and increased leukocyte adhesion. ECM remodeling involves the overproduction of type I collagen and the activation of MMPs, resulting in vessel stiffening and a loss of elasticity. VSMC dysfunction, marked by phase transitions and apoptosis regulated by TGF-β and ROS, further drives fibrosis. The activation of ACE and Angiotensin II receptors intensifies oxidative stress, inflammation, and fibrosis. Rarefaction, characterized by a loss of capillary density, reduces blood flow and oxygen delivery, contributing to elevated vascular resistance and impaired microvascular function. These interconnected mechanisms underline the progression of hypertensive vascular disease.

## Figures and Tables

**Table 1:** Key features of microvascular dysfunction in hypertension.

Aspect	Key Characteristics	Pathophysiological Consequences
Oxidative Stress	- Excessive ROS production (via NADPH oxidase, mitochondrial dysfunction, eNOS uncoupling)	- Reduced NO bioavailability - Impaired vasodilation - Endothelial dysfunction
Endothelial Dysfunction	- Decreased NO synthesis - Peroxynitrite (ONOO^−^) formation	- Loss of vascular homeostasis - Increased vascular resistance
Inflammatory Response	- NF-κB activation - Upregulation of cytokines, chemokines, and adhesion molecules	- Endothelial activation - Leukocyte adhesion and transmigration
Fibrosis and ECM Remodeling	- Type I collagen overproduction - MMP activation and ECM degradation	- Vessel stiffening - Reduced microvascular elasticity
VSMC Dysfunction	- Phase transition of VSMCs - Apoptosis mediated by TGF-β and ROS	- Loss of contractility - Fibrotic changes in vessel walls
Angiotensin II Activation	- ACE and Angiotensin II receptor signaling	- Exacerbation of ROS production - Promotion of inflammation and fibrosis
Rarefaction	- Loss of capillary density due to apoptosis and impaired angiogenesis	- Reduced blood flow and oxygen delivery - Increased peripheral resistance

ROS: Reactive Oxygen Species; NADPH: Nicotinamide Adenine Dinucleotide Phosphate; eNOS: Endothelial Nitric Oxide Synthase; NO: Nitric Oxide; ONOO^−^: Peroxynitrite; NF-κB: Nuclear Factor kappa B; ECM: Extracellular Matrix; VSMC: Vascular Smooth Muscle Cell; MMP: Matrix Metalloproteinase; TGF-β: Transforming Growth Factor-beta; ACE: Angiotensin-Converting Enzyme.
